# Perceptibility and Acceptability Thresholds for Color Differences in Ceramic Shade Tabs: A Comparison Between Dentists and Patients

**DOI:** 10.4317/jced.62287

**Published:** 2024-12-01

**Authors:** Vanessa Schussler, Daniel Yuydi Kawakami, Marco Antonio Garcia Rocha, Mario Alexandre Coelho Sinhoreti, Mateus Garcia Rocha, Dayane Oliveira

**Affiliations:** 1 D.D.S, M.S. Candidate. Department of Restorative Dental Sciences, University of Florida, College of Dentistry, 1395 Center Drive, Gainesville, FL, United States; 2 D.D.S, Alumni, Dentist. Department of Restorative Dentistry, Piracicaba Dental School, State University of Campinas, Av. Limeira 901, Piracicaba, SP, Brazil; 3 D.D.S, Dentist, Research Volunteer. Department of Restorative Dental Sciences, University of Florida, College of Dentistry, 1395 Center Drive, Gainesville, FL, United States; 4 D.D.S., M.S., Ph.D, Full Professor. Department of Restorative Dentistry, Piracicaba Dental School, State University of Campinas, Av. Limeira 901, Piracicaba, SP, Brazil; 5 D.D.S., M.S., Ph.D, Clinical Associate Professor. Department of Restorative Dental Sciences, University of Florida, College of Dentistry, 1395 Center Drive, Gainesville, FL, United States; 6 D.D.S., M.S., Ph.D. Clinical Associate Professor. Department of Restorative Dental Sciences, University of Florida, College of Dentistry, 1395 Center Drive, College of Dentistry, Gainesville, FL, United States

## Abstract

**Background:**

The aim of this study was to evaluate the 50%:50% perceptibility thresholds (PT) and acceptability thresholds (AT) for color differences in ceramic shade tabs observed by dentists and patients using CIEDE2000 color difference formula.

**Material and Methods:**

Twenty-two combinations of ceramic shade tabs from the VITA 3D Master shade guide were assembled to be used for the visual comparison analyses. The color difference between each shade tab pair was numerically determined by spectrophotometry using the VITA EasyShade V, and calculated using the CIEDE2000 formula (ΔE00). Twenty dentists and twenty patients were recruited for this study. All participants performed the visual assessment of the provided shade tab pairs under D65 illumination and a grey background, and requested to determine if they could perceive a color difference between them (PT) and whether they considered the combination clinically acceptable (AT). The correlation between numeric data of color difference between the shade tabs, and the perceptibility and acceptability thresholds given by the participants was analyzed by logistic regression (α=0.05; β=0.0085).

**Results:**

The PT for dentists was ΔE00= 2.29, and ΔE00= 2.27 for patients. The AT for dentists was ΔE00=2,41, and ΔE00=2,83 for patients. The results showed a statistically significant difference between PT and AT thresholds for both dentists and patients. However, there was no statistically significant differences in PT (*p*=0.39) or AT (*p*=0.54) between patients and dentists.

**Conclusions:**

Within the limitations of this study, it was possible to conclude that while the PT and AT vary significantly within each group, they are statistically similar between dentists and patients when discriminating color differences in ceramic tabs.

** Key words:**Color difference, esthetic, aesthetic, spectrophotometry, CIEDE2000.

## Introduction

Colorimetry is the science that involves the quantification and physical investigation of the phenomenon of color perception. Color perception is a psycho-visual process in which the color is perceived by the eyes and interpreted by the brain (1,2). For this reason, often, the numerical data found in complex colorimetric analyzes are not always actually perceived by the eye and/or the human brain (3).

The retina of the human eye has three types of color-sensitive cells, the cones. They can be sensitive to different wavelengths, such as the long waves in the red spectrum, the medium waves in the green spectrum, and the short wavelength within the visible blue spectrum. For this reason, cones are often referred to as red, green, or blue, according to the visible spectrum of the color to which it is sensitive (1).

However, after sensing the cones, the brain’s interpretation of color is subjective. In 2005, neuroscientists at the University of Rochester found that the number of cones present in the human retina is highly variable; however, people can perceive colors similarly. This study highlights the brain’s strong influence on the interpretation of color initiated by the human eye’s perception (4).

Due to the subjectivity of color classification by visual perception, the use of electronic devices such as spectroradiometers and spectrophotometers is indicated in research to evaluate and measure color differences objectively (5,6). Spectrophotometry processes light reflection data through color parameters in the CIELab space. The CIELab color space is a color-opponent space with three axes: L for lightness, and a and b for the color-opponent dimensions. Where a represents the position between green and red, and b represents the position between blue and yellow. It was created by the International Commission on Illumination (CIE) and is designed to be perceptually uniform, meaning that the same amount of numerical change in these values corresponds to roughly the same amount of visually perceived change (7,8).

Visual thresholds for color discrimination have long been an essential quality control tool in various industries. In dentistry, these thresholds serve as valuable resources for quality control in selecting and evaluating dental materials, interpreting color-related findings (both visual and instrumental) in clinical practice and research, and ensuring standardization within the field (9).

Visual thresholds consist of two main concepts: the perceptibility threshold (PT) and the acceptability threshold (AT). The perceptibility threshold is when a person visually notices a color difference between two objects, while the acceptability threshold is the point at which this color mismatch remains clinically acceptable. In research, they are usually expressed through their 50%:50% values, which is when 50% of the participants can perceive that difference and 50% cannot (5,9).

The study by Paravina *et al*. (2015) evaluated PT and AT in Dentists, auxiliaries, technicians and dental students (10). This study included laypersons to represent patients, and they are an important group to consider in threshold studies, since they are actively making decisions about their treatment together with the dental team, and they should be included in the decision-making process about the color and appearance of their restorations. The color of an anterior restoration is the most significant factor when patients assess the quality of the dental work, particularly in the anterior teeth (3,11)

Several published papers have explored the topic of color threshold in Dentistry (9,10,12-20). In a recent review paper, values of PT and AT for teeth and tooth-colored materials were set at 0.8 and 1.8 ΔΕ00 units, respectively (19) Threshold research is very complex, and the dental professionals’ limited color science expertise, has led to suboptimal study designs and data processing in some cases, undermining the credibility of certain findings. There is a lack of systematic approach and standardized research methods, leading to an inconsistency in the results. Concerns include the selection and number of observers, uncontrolled shade matching conditions and methods, and discrepancies between the visually observed area and the area measured by instruments (9,20).

This paper aimed to evaluate the 50%:50% perceptibility thresholds (PT) and 50%:50% acceptability thresholds (AT) for color differences in ceramic shade tabs observed by dentists and patients using CIEDE2000 color difference formula. The null hypotheses were that: 1) There would be no differences between the PT and AT, and 2) There would be no differences in PT and AT among dentists and patients.

## Material and Methods

-Shade tabs preparation

Five VITA 3D Master shade guides (VITA, Bad Sackingen, Germany) were used in this study. First, possible color differences between similar shade tabs from the different shade guides were measured using a spectrophotometer (see methodology used in the following section). After confirming there were no significant differences in color among similar shade tabs, twenty-two combinations of shade tabs were assembled to be used for the visual comparison analyses ( Table 1). All shade tabs color classification (egg. 1M1, 2M2, etc.) were hidden to prevent their identification. The shade tab combinations included 80% of the ceramics in different colors, while 20% of the pairs consisted of identical color tabs (control).

-Color difference assessment between shade tabs 

**Figure 1:**
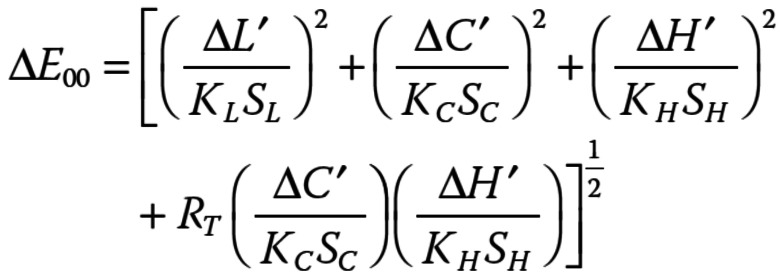
Formula.

The color differences between the shade tabs were measured through spectrophotometry analysis. First, color readings of each shade tab were performed under a grey background and D65 illumination using a pre-calibrated spectrophotometer (EasyShade V, Vita, Bad Sackingen, Germany) to obtain the numerical color data, according to the CIE Lab system. A clear PVS jig was used to standardize the positioning of the spectrophotometer tip on the middle third surface of the different shade tabs, so the reading would be performed in the same area of the different shade tabs. The color differences between the tabs were numerically calculated using the CIEDE2000 formula (CIE, 2018) (8): (Fig. 1).

Where ∆L′, ∆C′, and ∆H′ are the differences in value, chroma, and hue. RT is an interaction function for the interaction between chroma and hue differences in the blue region. SL, SC, and SH are weighting functions which adjust the total color difference for variation in the location of the color difference pair in L′, a′, and b′. KL, KC, and KH are parametric factors to correct experimental conditions (21), and were all set to 1.0, under reference conditions determined by the CIE technical report (8).

-Recruitment of volunteers 

This clinical study was approved by the local Institutional Review Board (IRB) (CAAE: 66758717.0.0000.5418; # 2.185.864). This clinical study was performed at Piracicaba Dental School, State University of Campinas, Piracicaba, SP, Brazil. Volunteers were recruited and selected based on the following eligibility criteria in order to enroll at least 40 volunteers in the study, those being at least 20 dentists, and 20 patients. The individuals included in this study were 18-35 years old with normal color vision who passed a dental color matching competency test (further described below).

All the recruited volunteers were subjected to the Ishihara color vision test (38 plates edition) with help and counseling of an ophthalmologist (22). The volunteers that were deemed to have normal color vision were requested to take a dental color matching competency test according to ISO11644-2. The subjects were requested to pair shade tabs from two VITA Classical shade guides with original markings covered and have correctly assigned a minimum of 75% of the samples.

-Color perceptibility and acceptability thresholds 

A total of 20 dentists and 20 patients were enrolled in this study. The participants were requested to identify color differences in between the 22 shade tab pairs selected. Visual color differences analyses were performed under standardized viewing conditions (windowless completely dark room, viewing booth with neutral gray background, 45o angulation, D65 illuminant, CRI ≥ 90; illuminance of 1000 lx, diffuse/0° optical geometry, at a viewing distance of 30cm) according to ISO 28642:2016 (5) and ISO 7491:2000 (23). The volunteers were given the time for visual adaptation to darkness before starting. All shade tab pairs were presented in the same order to all observers.

The frequency level of perceived/accepted color differences was determined and recorded in an answering sheet. Participants were requested to verify the perception of color difference between each shade tab pairs (PT), and if so whether it was clinically acceptable (AT) or not. For this, participants were requested to answer yes or no to the following questions: “Do you see a difference in color between the two tabs?” and “Do you consider this difference in color acceptable in a patient’s mouth?” 

-Tabulation and analysis of data 

The correlation between the numerical data of color difference, and the perceptibility and acceptability thresholds reported by dentists and patients were submitted to a univariate logistic regression model where the response variable was perception, or not; acceptance, or not; of the color difference between the different shade tab pairs, identifying the ∆E00 most adjusted for PT and AT for dentists and patients. The model was adjusted to an alpha significance level of 0.5 and beta of 0.2, with a threshold for logistic regression of 66.6%, following that suggested by ISO/TR 28642:2016 (5) and ISO 7491:2000 (23).

## Results

**Figure 2:**
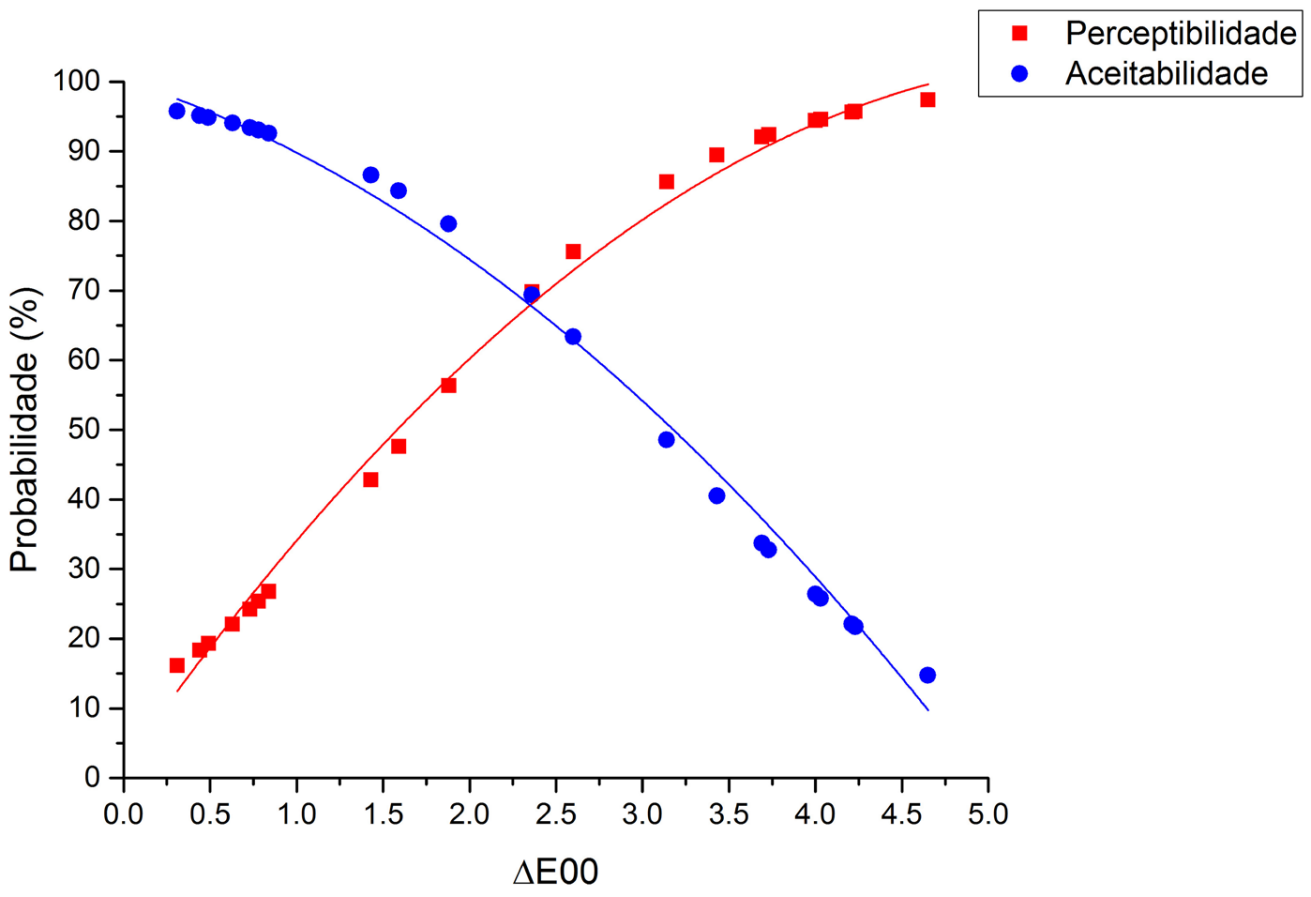
Logistic regression results regarding the numerical data of color difference and the probability of perceptibility and acceptability thresholds for dentists.

**Figure 3:**
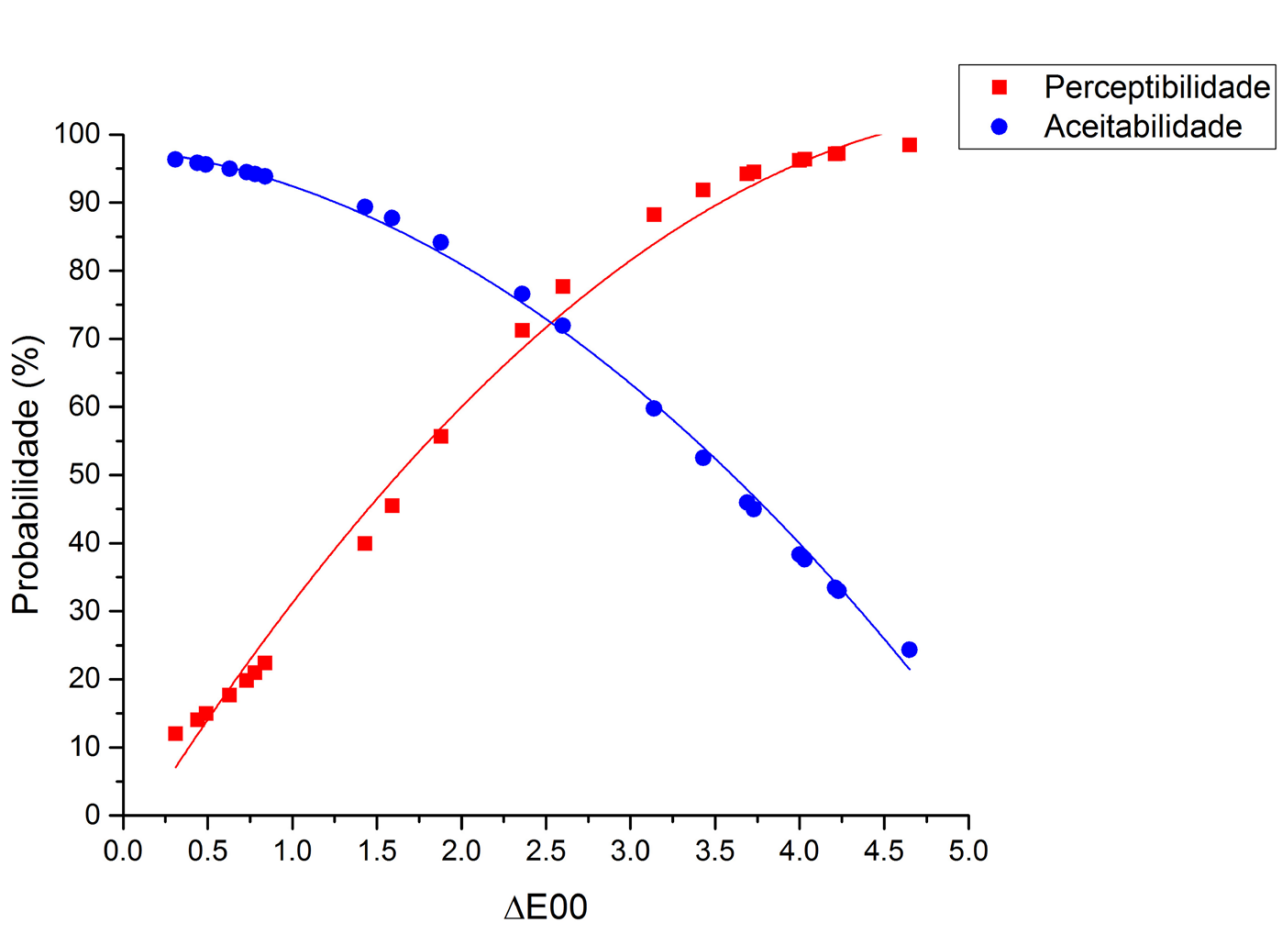
Logistic regression results regarding the numerical data of color difference and the probability of perceptibility and acceptability thresholds for patients.

 Table 1 shows the numerical color differences (CIEDE2000) between the different shade tab pairs and their respective PT and AT from the visual color analysis by dentists and patients. Figures 2 and 3 illustrate the logistic regression results regarding the CIEDE2000 color differences and the probability of perceptibility and acceptability of that color difference for dentists and patients, respectively. As it can be observed from the results of this study, the 50%:50% PT for dentists was ΔE00 = 2.29 (OR 3.36 ± 0.46, 95% CI 2.57-4.40, *p*<0.001), white AT was ΔE00 = 2.41 (OR 0.32 ± 0.40, 95% CI 0.25-0.41, *p*<0.001). For the patients, the 50%:50% PT was ΔE00 = 2.27 (OR 4.11 ± 0.59, 95% CI 3.10-5.46, *p*<0.001) and the AT was ΔE00 = 2.83 (OR 0.36 ± 0.41, 95% CI 0.29-0.45, *p*<0.001). The results demonstrated a statistically significant difference between PT and AT thresholds for both dentists and patients; but there were no statistically significant differences in PT (*p*=0.39) or AT (*p*=0.54) between patients and dentists.

## Discussion

The objective of this study was to evaluate the 50%:50% perceptibility thresholds (PT) and 50%:50% acceptability thresholds (AT) for color differences in ceramic shade tabs observed by dentists and patients using CIEDE2000 color difference formula. The first null hypothesis that there would be no differences between the PT and AT was rejected, as the results demonstrated a statistically significant difference between PT and AT thresholds for both dentists and patients. On the other hand, the second null hypothesis that there would be no differences in PT and AT among dentists and patients was accepted as there was no statistically significant differences in PT (*p*=0.39) or AT (*p*=0.54) between patients and dentists. 

As previously described, color perception is a psycho-visual process in which color is perceived by the human retina’s cones and interpreted by the brain (1,2). Although the number of cones present in the human retina is highly variable, people can perceive colors in a similar way (4). In fact, as observed in this study, the color differences are perceived similarly by both dentists and patients, that is, regardless of the degree of training or instruction in the subject. It is important to point out that in this study there was an equal number of women and men as participants in both groups (dentists and patients), and all participants had normal color vision. Color blindness, also known as color vision deficiency (CVD), is a condition in which an individual has difficulty distinguishing between certain colors. This condition affects color perception because it is caused by an abnormality in the retinal photoreceptors called cones, which are responsible for detecting colors. In men the prevalence of color blindness is around 8%, meaning 1 in 12 men are color blind; and in women, the prevalence is 0.5% (24). There are three types of cones, each sensitive to a specific range of wavelengths: red, green, and blue. In individuals with normal color vision, these three types of cones work together to perceive a wide range of colors. However, in those with color blindness, one or more of these cone types are either missing, non-functioning, or detect a different color than normal. As a result of these abnormalities in the cones, people with color blindness have a limited color perception compared to those with normal color vision.

Moreover, all participants in this study were submitted to a dental color matching competency test. The International Organization for Standardization (ISO) requires a dental color matching competency test as part of the ISO/TR 28642:2016 (5) standard, which provides guidance on the assessment of color in dentistry. The purpose of this requirement is to ensure that individuals involved in color matching tasks have the competency to achieve accurate and consistent results.

Individual factors like age and visual acuity can also affect how color is perceived. This happens for several reasons such as 1- yellowing and opacity of the lens with age leading to changes in color perception, particularly in the blue-yellow range; 2- decreased sensitivity of cone cells, leading to a reduced ability to distinguish between different colors; 3- pupil reduction in size, that reduces the amount of light entering the eyes and affecting color perception, especially between subtle color differences; and 4- other age-related conditions, such as cataracts, glaucoma and macular degeneration for this same reason (25).

It is known that cultural influences can shape how people associate colors with social status or emotional states. These associations can vary widely across different cultural groups, underscoring the complexity and subjectivity of color perception and its acceptability (26). Previous studies (10;27) demonstrated that patients tend to accept greater color changes concerning natural teeth compared to dentists. Perez *et al*. (2019) found that 50%/50% whiteness AT among dentists and laypersons are at 0.72 and 2.60 respectively (12). Paravina *et al*. (2015) found a similar PT by patients and dentists with values between 0.6 and 1.0. Regarding the results of AT, they found thresholds of 1.8 for dentists and 2.0 for patients (10). In this study, the 50%:50% PT for dentists was 2.29, while AT was 2.41; and for the patients, PT was 2.27 and AT was 2.83. However, it is difficult to compare these types of studies because the results will vary according to many factors including the type of population being studied, the formula and thresholds being used, as well as the specimen’s material used, and viewing conditions for the color difference discrimination (17;20;28-29).

In this study, although there was statistically significant difference between PT and AT thresholds for both dentists and patients; there was no statistically significant differences in PT (*p*=0.39) or AT (*p*=0.54) between patients and dentists. Still, it is important to recognize patients’ active role in treatment decisions alongside the dental team. It is essential that they are included in the decision-making process regarding the color and appearance of their restorations. A study showed that the highest disagreement among dentists and patients regarding anterior prosthetic restorations the highest the dissatisfaction with the natural appearance and color of those restorations at a rate of 52.2% (30).

It is important to point out that although this study followed all standard requirements and guidelines to ensure reliable results, since observer repeatability and reproducibility in color-related studies are very hard to achieve, this study used ceramic shade tabs as specimens. This can be disclosed as a limitation of this study as color difference discrimination will be different for different materials due to their distinct optical properties and characteristics (5,19). Moreover, research on visual thresholds indicates that differences should be meticulously designed, encompassing a range of color differences and increments. These should include both differences close to zero and those that are clearly visually unaccepTable (5,19). In the present study, lightness differences were designed to include light, medium, and dark colors, as well as combinations with an exact match and markedly different colors.

According to Khashayar, *et al*., the most referred and cited literature on this subject is from the 80’s, which is surprising considering the increase in esthetic demands from patients and dental providers in our current society. It was to be expected to find more current literature about this subject. Still, this same article concludes that most clinical studies refer to PT and AT extracted from a few literature examples that were published more than thirty years ago and that not necessarily used a similar material (ceramic, composite, tooth, etc) (29).

The dental literature clearly lacks consensus on the extent of color difference that constitutes an accepTable shade mismatch or what is considered perceivable to observers. PT and AT values used as a refence by these studies have been extracted from different articles with different study designs (29). However, high quality research about this subject has a high importance in the field of dentistry. Relevant topics in this area include color stability of materials, quality control of different batches, interaction between different materials regarding color, assessment of color match between restorations and natural teeth, or between different types of restorations. It would be interesting to expand the study of this topic with a larger sample size, larger and more diverse evaluator groups, inclusion of different materials such as composites and natural teeth, and finally attempting an *in-vivo* study design. However, within the limitations of this study it was possible to conclude that while the PT and AT vary significantly within each group, they were statistically similar between dentists and patients when discriminating color differences in ceramic tabs.

## Figures and Tables

**Table 1:** CIEDE2000, PT and AT from the Visual Color Evaluation by Dentists and Patients.

Ceramic Pair	Shade 1	Shade 2	ΔE00	Dentist			Patient		
				Color Evaluation			Color Evaluation		
				Perceptible		Not Perceptible	Perceptible		Not Perceptible
				Acceptable	Not Acceptable		Acceptable	Not Acceptable	
1	2M1	2M1	0,63 (0,18)	10%	0%	90%	17%	0%	83%
2	2M2	2M1	2,60 (0,09)	90%	10%	0%	42%	33%	25%
3	2M2	2M3	3,69 (0,30)	10%	80%	10%	25%	75%	0%
4	3M1	3M1	1,59 (0,16)	0%	0%	100%	0%	0%	100%
5	3M1	3M2	4,65 (0,39)	10%	90%	0%	42%	58%	0%
6	3M3	3M2	4,03 (0,27)	30%	70%	0%	50%	50%	0%
7	3M3	3M3	0,44 (0,31)	30%	0%	70%	25%	0%	75%
8	4M1	4M1	0,84 (0,23)	30%	20%	50%	33%	0%	67%
9	4M1	4M2	4,21 (0,20)	0%	100%	0%	17%	83%	0%
10	4M2	4M3	3,43 (0,11)	10%	80%	10%	33%	67%	0%
11	5M1	5M2	4,23 (0,13)	0%	80%	10%	33%	67%	0%
12	5M2	5M3	3,73 (0,15)	0%	100%	0%	25%	75%	0%
13	5M3	5M3	0,49 (0,11)	20%	10%	70%	0%	17%	85%
14	1M1	1M1	0,78 (0,23)	30%	0%	70%	8%	8%	83%
15	2M1	3M1	4,00 (0,36)	70%	20%	10%	58%	25%	17%
16	4M1	5M1	4,65 (0,13)	30%	70%	0%	50%	50%	0%
17	4R1.5	4R1.5	0,31 (0,07)	10%	90%	0%	17%	8%	75%
18	4M1	4L1.5	3,14 (0,39)	40%	60%	0%	33%	68%	0%
19	3M1	3R1.5	2,36 (0,28)	40%	60%	0%	42%	58%	0%
20	3L1.5	3L1.5	0,73 (0,33)	0%	10%	90%	25%	0%	75%
21	2R1.5	2M1	1,88 (0,05)	0%	0%	100%	25%	8%	67%
22	2L1.5	2L1.5	1,43 (0,16)	30%	0%	70%	17%	0%	83%

## Data Availability

The datasets used and/or analyzed during the current study are available from the corresponding author.
